# Long-Term Organic Farming Manipulated Rhizospheric Microbiome and *Bacillus* Antagonism Against Pepper Blight (*Phytophthora capsici*)

**DOI:** 10.3389/fmicb.2019.00342

**Published:** 2019-02-27

**Authors:** Huixiu Li, Xiaoxu Cai, Jingyang Gong, Ting Xu, Guo-chun Ding, Ji Li

**Affiliations:** ^1^College of Resources and Environmental Sciences, China Agricultural University, Beijing, China; ^2^Beijing Key Laboratory of Biodiversity and Organic Farming, Beijing, China

**Keywords:** organic farming, disease-suppressive soil, soil microbiome, *Capsicum annuum* L., *Phytophthora capsici*, rhizosphere, *Bacillus*

## Abstract

Soil-borne diseases are often less severe in organic farms, possibly because of the recruitment of beneficial microorganisms by crops. Here, the suppressiveness of organic, integrated, and conventionally managed soils to pepper blight (*Phytophthora capsici*) was studied in growth chamber experiments. Disease incidence was 41.3 and 34.1% lower in the soil from an organic farming system than in either the soil from the integrated or from the conventional farming systems, respectively. Beta-diversity of rhizospheric microbial communities differed among treatments, with enrichment of *Bacillus, Sporosarcina*, Acidobacteria *Gp5, Gp6*, *Gp22*, and *Ignavibacterium* by the organic soil. Cultivation-dependent analysis indicated that 50.3% of *in vitro* antagonists of *P. capsici* isolated from the rhizosphere of healthy peppers were affiliated to *Bacillus*. An integration of *in vitro* antagonists and bacterial diversity analyses indicated that *Bacillus* antagonists were higher in the rhizosphere of pepper treated by the organic soil. A microbial consortium of 18 *in vitro Bacillus* antagonists significantly increased the suppressiveness of soil from the integrated farming system against pepper blight. Overall, the soil microbiome under the long-term organic farming system was more suppressive to pepper blight, possibly owing to *Bacillus* antagonism in the rhizosphere. This study provided insights into microbiome management for disease suppression under greenhouse conditions.

## Introduction

Increasing evidence suggests that soil microbiomes, or those associated with crops, play key roles on plant health (Gómez Expósito et al., 2017). Bacterial diversity studies indicate that taxonomic groups such as Bacteroidetes (Yin et al., 2013; Xiong et al., 2015), Actinobacteria (Mendes et al., 2011; Xiong et al., 2015, 2017; Cha et al., 2016; van der Voort et al., 2016), Acidobacteria (Shen et al., 2015; van der Voort et al., 2016; Xiong et al., 2017), Firmicutes (Mendes et al., 2011; Shen et al., 2015; Xiong et al., 2015, 2017), and Proteobacteria (Mendes et al., 2011; Michelsen et al., 2015) are possibly involved in the suppression of these plant diseases. Moreover, *Bacillus* has been reported as an important plant growth-promoting bacteria (Fan et al., 2018). Severities of soil-borne plant diseases, such as those caused by *Rhizoctonia solani* (Postma et al., 2008), *Verticillium albo-atrum* (Postma et al., 2008), *Phytophthora infestans* (Wang et al., 2001), and *Fusarium* (Champeil et al., 2004; Lenc et al., 2015) in organic farms, have often reportedly been lower than in conventional farms. In organic farms, a large amount of organic fertilizer may contribute to the development of disease suppressiveness, often referred to as general suppressiveness, by triggering activities of soil indigenous microorganisms non-specifically (Qiu et al., 2012; Wang et al., 2015; Liu et al., 2018). Thus, general suppressiveness was not believed to be attributed to a few taxonomic groups as much as to specific disease-suppressive soil (Weller et al., 2002). However, long-term application of organic fertilizer could cause profound changes on soil microbial communities, as evidenced in both field and greenhouse experiments (Marschner et al., 2003; Ndubuisi-Nnaji et al., 2011; Hartmann et al., 2014; Luo et al., 2015), and some alteration of microbial communities was indicated to be associated with disease suppression (Zhang et al., 2017). Meanwhile, changes in soil physicochemical properties, such as organic matter and nutrient content, were accompanied by shifts in soil microbiomes (Fernandez et al., 2016; Li et al., 2017; Pronk et al., 2017). Alterations in physicochemical properties likely shape biogeochemical interfaces in the soil where microorganisms live; thus, such changes may also affect the activities and fate of both phytopathogens (Strunnikova et al., 2015) and other microorganisms (Pronk et al., 2017). Overall, it is still unclear whether, or to what extent, changes in soil microbiome after long-term organic farming may contribute to soil-borne disease suppressiveness.

A long-term greenhouse experiment was set up in 2002 at Quzhou experimental station, in Hebei Province, China. This experiment included organic, integrated, and conventional farming systems for evaluation of soil suppressiveness to pepper blight. All three farming systems were under the same schemes of crop rotation, irrigation, and tillage, but they differed in fertilization and plant protection management. Differences in both microbial structure and physicochemical properties (Han et al., 2017) were detected among these different farming systems. Disease incidence of downy mildew, leaf mold, early and late blight, powdery mildew, and bacterial angular leaf spot on tomato (*Solanum lycopersicum* L.) or cucumber (*Cucumis sativus* L.) were often lower in the organic farming system than in the conventional farming system (Yang et al., 2009a,b). This long-term experiment provided an opportunity to explore the mechanisms underlying organic soil effects on plant health that depend on soil microbiomes forged by long-term organic farming.

Here, we set up a growth chamber experiment under controlled conditions to evaluate the effects of the soil microbiome from organic, integrated, and conventional farming systems on the suppression of pepper blight disease, a major disease caused by *Phytophthora capsici* that is responsible for worldwide losses of over US $100 million in pepper alone (Barchenger et al., 2017). To mitigate the potential effects of soil physicochemical properties, pepper plants were grown in sand and soil mixed at a ratio of 5:1.2 (v:v). Rhizospheric microbiomes under different treatments were studied intercross by cultivation-dependent and high throughput sequencing analysis. In this study, our specific questions were as follows: (1) Whether and to what extent the soil microbiome forged by long-term organic farming could suppress pepper blight disease; (2) whether different soil microbiome inoculation treatments affect the assemblage of rhizospheric microbial community of pepper; and (3) what taxa are associated with the suppression of pepper blight disease in the soil managed organically?

## Materials and Methods

### Long-Term Organic Farming Experiment, Sampling, and Bioassay Experiment

Soil samples were collected from the long-term greenhouse experiment that was set up in 2002 at the Quzhou Experiment Station (36° 52′ N, 115° 01′ E), Hebei, China. Treatments included organic, integrated, and conventional farming systems. Details on the agricultural management in each case were as described by Han et al. (2017). Briefly, all farming systems followed the same schemes of crop rotation, irrigation, and tillage. The organic farming system was characterized by the application of compost (165 t ha^−1^ year^−1^) from chicken and cow manure, as well as biological and physical methods of plant protection (sticky yellow paper traps and insect net to control pests; Kocide containing copper hydroxide, mechanical removal of diseased plants and ridging planting to control plant diseases; mechanical/mulch to suppress weeds). Conventional farming followed the local farming style for greenhouse vegetable production and used chemical fertilizers (urea, calcium superphosphate, and potassium chloride), pesticides (Dimethomorph, Lambda-cyhalothrin, Imidacloprid and Carbendazim) and 46.8 t ha^−1^ year^−1^ of chicken and cow manure. Lastly, the integrated farming system used half the amount of chemical fertilizers and pesticides used in the conventional farming system and half the amount of organic fertilizer used in the organic farming system. For each farming system, a total of 75 soil cores (2 cm in diameter) from the soil’s top layer (1–20 cm depth) were sampled on June 2017. All soil samples were passed through a 2-mm mesh to remove stones and plant debris. Sieved samples were kept at 4°C prior to the growth chamber experiment (Ding et al., 2010).

The bioassay was performed as follows: “Cayenne” pepper (*C. annuum* L.) seeds (Zhong liang xin) were sown in pots (12 cm in diameter and 15 cm high) containing a mixture of 500 g of sterilized sand and 120 g soil, and grown for 28 days in a growth chamber (Hangzhou Lvbo Instrument Co., Ltd., LB-1000D-LED) at 30°C, 95% relative humidity and under a 12 h light (15000 lx) period. All plants were watered twice with standard Hoagland solution. The soil was used as an inoculant for the microbiome. All pots were completely randomized inside the growth chamber. Six seedlings per pot were kept up to 20 days after sowing; eight out of 20 pots were randomly selected for the challenge by *P. capsici. P. capsici* zoospores were cultured and prepared as previously described (Bi et al., 2014). 6 mL of zoospore suspension (approximately 5 × 10^5^ zoospores/mL) was dipped near the root of each seedling. Disease incidence was evaluated 7 days after inoculation. In parallel, four rhizosphere samplings of uninoculated seedlings were performed at 27, 31, 35, and 38 days after sowing (T1, T2, T3, and T4, respectively). Three pots were taken and all six seedlings in each pot were used as a replicate for each treatment and sampling. Rhizosphere samples were taken as follows: pepper plants were carefully removed from the pots and shaken to free the roots from as much soil as possible, and only those sand or soil particles strongly adhering to the roots remained adhered to the roots. Next, roots were washed vigorously with 0.85% NaCl solution. After removing root systems, the mixture was centrifuged at 6000 × *g* for five minutes. The pellet was kept at −20°C for total community DNA-extraction. Rhizosphere samples of plants free of disease symptoms 9 days after *P. capsici* inoculation were collected separately (Bi et al., 2014). A fraction of rhizosphere samples from healthy peppers were mixed with an equal volume of 40% glycerol and stored at −80°C immediately.

### High Throughput Sequencing Analysis of Bacterial 16S *rRNA* Gene Amplicon

Total microbial community DNA from rhizosphere samples was extracted using a FastDNA spin Kit for soil (MP, Biomedicals, Santa Ana, Carlsbad, CA, United States) according to the instructions by the manufacturer. High throughput sequencing analysis was performed using the platform of Hiseq2500. Briefly, universal primers 515F (5′-GTGCCAGCMGCCGCGGTAA-3′) and 909R (5′- CCCCGYCAATTCMTTTRAGT −3′) with a 12 nt unique barcode (Caporaso et al., 2010) were used to amplify 16S *rRNA* gene fragments. All sequence reads were trimmed and assigned to each sample based on barcodes. High quality sequences (length > 300 bp, without ambiguous base “N,” and average base quality score > 30) were used for downstream analyses. Generation of the taxonomic OTU was performed as previously described using a suite of public databases and software (Schloss et al., 2009; Caporaso et al., 2010; Ding et al., 2012; Cole et al., 2014; Yilmaz et al., 2014; Rognes et al., 2016). Comparisons of community composition, identification of taxa with significantly different relative abundance and network analysis were performed as previously described (Kropf et al., 2004; Hothorn et al., 2017). A co-occurrence network was constructed using Spearman’s rank correlation coefficient (cor > 0.6, *p* < 0.01) (Barberán et al., 2012). This network was analyzed using the gephi (version 0.91) software (Bastian et al., 2009). Community composition was visualized by principle coordinate analysis (PCoA) based on pairwise Bray-Curtis distance calculated from the relative abundance of different OTUs. All statistical analyses and plotting were performed with the R 3.1.2^1^ software, and these tools have been implemented into a galaxy instance ^2^ according to the description by the galaxy developing team^3^. All sequences were submitted to the NCBI SRA (PRJNA497732).

### Isolation, Screening, BOX-PCR, and 16S *rRNA* Sequencing Analysis of *in vitro* Antagonists of *P. capsici*

Serial dilutions of rhizosphere samples from pepper plants free of disease symptoms were plated on R2A medium (Beijing Land Bridge Technology Co., Ltd.) supplemented with 100 mg/L cycloheximide, followed by incubation at 30°C for 2 to 3 days. All colonies with different morphologies were selected for further analyses. *In vitro* antagonists against *P. capsici* were screened according to Yang et al. (2012). Genomic DNA of antagonists was extracted using a bacterial genomic DNA extraction kit (Beijing Biomed Co., Ltd.). BOX-PCR analysis was performed according to Adesina et al. (2007) using BOX-A1R primers (5′-CTACGGCAAGGCGACGCTGACG-3′) (Louws et al., 1994). Digital images were further analyzed by the software package GelCompar II (Applied Maths, Kortrijk, Belgium, China) to assign bacterial isolates into the different BOX-PCR patterns when profile similarity was less than 80%. For each BOX-PCR pattern, one isolate was randomly selected for Sanger sequencing analysis of the 16S *rRNA* gene amplified by primers 27F (5′-AGAGTTTGATCATGGCTCAG-3′) and 1492R (5′-TACGGTTACCTGTTACGACTT-3′) (Lane, 1991). A phylogenetic tree was constructed with software Mega 6.0 (Tamura et al., 2013). BOX-PCR was used for studying bacteria of the same species, of which 16S *rRNA* genes are highly similar. Here, subsequences between 515F and 909R were selected to identify unique phylotypes by the VSEARCH software (Rognes et al., 2016). These unique phylotypes were mapped against the 16S r*RNA* sequence library with a minimum sequence identity of 99% using a standalone BLASTN analysis.

### Biological Control Experiment Under Greenhouse Conditions

Eighteen *Bacillus* antagonists (11 *Bacillus methylotrophicus*, three *Bacillus licheniformis*, two *Bacillus aerophilus*, one *Bacillus cereus* and one *Bacillus subtilis*) isolated from healthy peppers grown in the mixture with soil from the organic farming system were selected to compose a microbial consortium whose ability to improve the suppressiveness of soil from integrated farming system to pepper blight was evaluated. The highest disease incidence was observed in the soil from the integrated farming system in two previous bioassays. *Bacillus* spores were prepared according to Paidhungat et al. (2000). When pepper seedlings were 20 days old, eight milliliters of the suspension containing *Bacillus* spores (approximately 2 × 10^8^ spores/mL) was inoculated. The challenge with *P. capsici* and the evaluation of disease incidence were performed according to the above description.

## Results

### Pepper Blight Was Less Severe in Plants Grown in an Organic Soil

**FIGURE 1:**
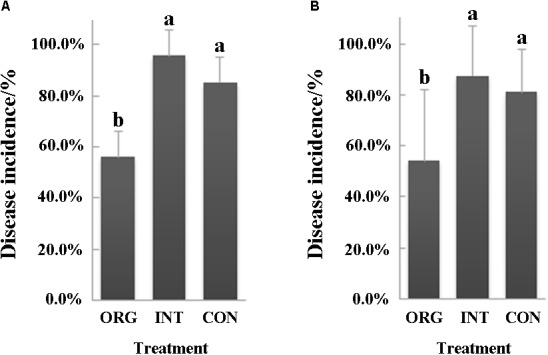
Percentage (mean ± SD, *N* = 8) of pepper seedlings showing *Phytophthora* blight (*P. capsici*) symptoms in soils from the organic (ORG), integrated (INT), and conventional (CON) farming systems (*p* < 0.05, Duncan’s multiple range test) in the first **(A)** and second **(B)** bioassay. Significant differences are indicated by different letters.

Two independent bioassay experiments with soils from organic, integrated, and conventional farming systems were performed to evaluate their suppressiveness to pepper blight caused by *P. capsici*. Both experiments demonstrated that soil from the organic farming system was more suppressive than either the integrated or the conventional farming system (Figure 1A,B). In the first bioassay, the percentage of disease incidence was 56.3% in the organic soil, which was 41.3 and 34.2% lower than in either the integrated or the conventional farming system, respectively (Figure 1A). Further, in the second bioassay, the percentages of disease incidence were 54.2% in the organic soil, 87.5% in the soil from the integrated farming system, and 81.3% in the soil from the conventional farming system (Figure 1B).

### Beta Diversity of Rhizospheric Microbial Communities Varied Among Soil Treatments

**FIGURE 2:**
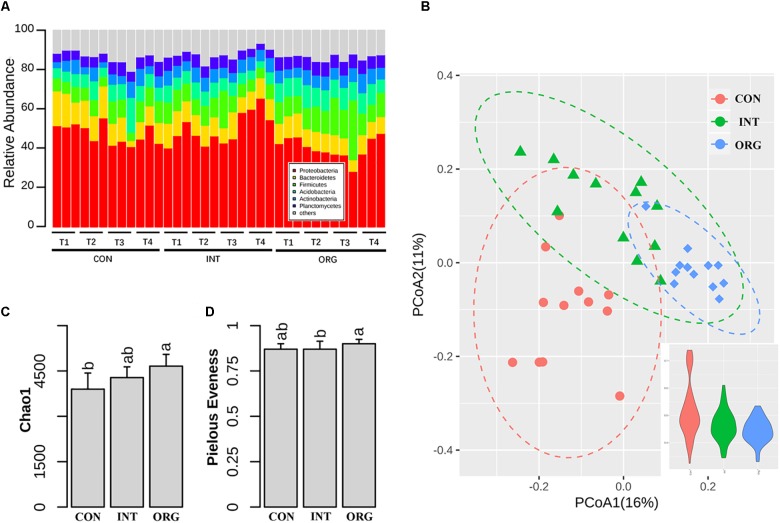
Relative abundance of different phylum **(A)**, PCoA analysis of microbial community and variation within treatments **(B)**, species richness index **(C)**, and evenness index **(D)** in the rhizosphere of pepper seedlings in soils from the organic (ORG), integrated (INT), and conventional (CON) farming systems during the four sampling periods, i.e., T1 (27 days after sowing), T2 (31 days after sowing), T3 (35 days after sowing), and T4 (38 days after sowing). Significant differences are indicated by different letters.

In all, 656,105 16S *rRNA* gene sequences were acquired from 36 rhizosphere samples collected at four samplings. Here, we excluded 60,322 sequences affiliated to *Chloroplast* or *Cyanobacteria* from the following analysis. More details on sequencing, such as number of reads, coverage, and unclassified taxa, are shown in Supplementary Table S1. For all samples, Proteobacteria (46%) was dominant, followed by Bacteroidetes (10.7%), Firmicutes (10.2%), Acidobacteria (8.1%) and Actinobacteria (6.1%) (Figure 2A). PCoA based on Bray-Curtis dissimilarities revealed that the rhizospheric bacterial community was different among treatments (Figure 2B), with more fluctuation detected by the soil in the conventional farming system (Figure 2B). Partition analysis of variation indicated that treatment and sampling explained 22% and 10% of total variation in microbial rhizospheric community, respectively. Permutation analysis also confirmed that community composition was significantly different among treatments (Supplementary Table S2). Chao1 richness in the rhizospheric bacterial community was slightly higher in treatment by the organic soils than that by soil from the conventional farming system (Figure 2C). Pielou’s evenness was slightly lower in treatment by the organic soil as compared to that by soil from the integrated farming system (Figure 2D).

### Taxa Associated With the Suppression of Pepper Blight

**FIGURE 3:**
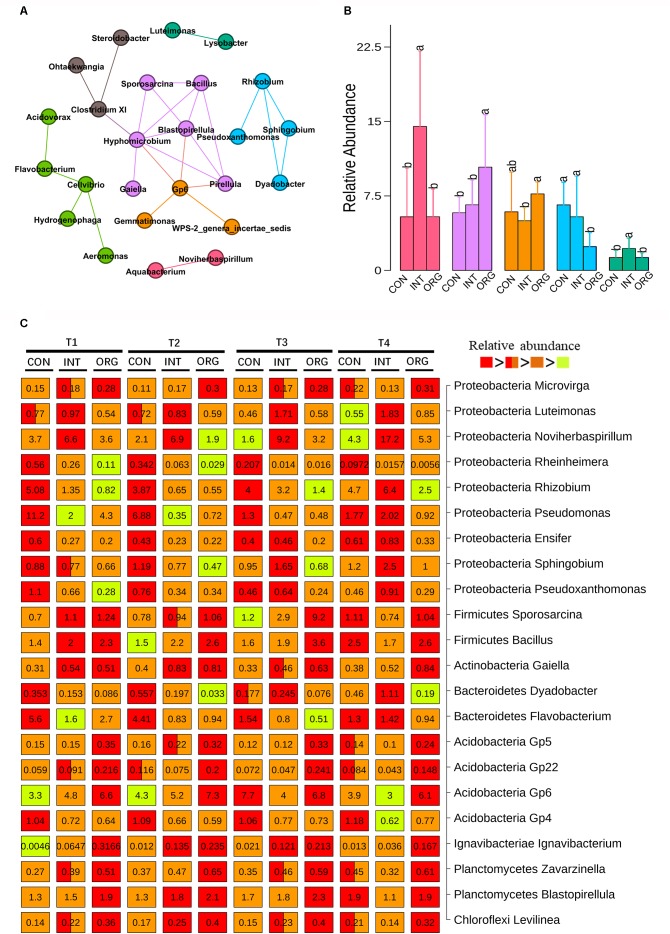
Microbial hubs **(A)** and their relative abundance **(B)** in the rhizosphere of pepper seedlings by soils from the organic (ORG), integrated (INT) and conventional (CON) farming systems at T1 (27 days after sowing), T2 (31 days after sowing), T3 (35 days after sowing), and T4 (38 days after sowing) stages as indicated by co-occurrence network analysis. Each color represents a different microbial hub and connections indicate significance (*p* < 0.001), Spearman’s rank correlation coefficient > 0.6, co-occurrence between the taxa. **(C)** Genera with significantly different relative abundance among different treatments at four sampling times. Numbers on the box indicate relative abundance expressed as a percentage. Significant difference is indicated by a different color. A box with two colors indicates no significant difference from other treatment containing one of the two colors. Significant differences are indicated by different letters.

Genera that significantly correlated (spearman correlation efficient > 0.6 and *p* < 0.001) with each other were subjected to network analysis. In all, 25 correlated genera formed seven microbial hubs (Figure 3A). The relative abundance of five hubs varied among different treatments (Figure 3B). The hub consisting of *Bacillus, Sporosarcina, Hyphomicrobium, Gaiella, Pirellula*, and *Blastopirellula* was significantly more abundant in the treatment involving the organic soil than the other two treatments, in contrast to the hub that included *Rhizobium, Sphingobium, Pseudoxanthomonas*, and *Dyadobacter* (Figure 3B). Two other hubs (one comprised *Aquabacterium* and *Noviherbaspirillum* and the other included *Luteimonas* and *Lysobacter*) were significantly more abundant in the treatment involving soil from the integrated farming system, in contrast to the hub consisting of Acidobacteria *Gp6, Gemmatimonas* and *WPS-2_genera_incertae_sedis* (Figure 3B). Multiple comparisons were performed to identify taxa with significantly different relative abundance among treatments (Figure 3C). Eleven genera (*Bacillus, Sporosarcina, Gaiella, Blastopirellula, Rhizobium, Sphingobium, Pseudoxanthomonas, Dyadobacter*, *Luteimonas, Noviherbaspirillum*, and Acidobacteria *Gp6*) showed similar patterns of their corresponding microbial hub (Figure 3C). In addition, the lowest relative abundance of other genera affiliated to Proteobacteria, such as *Rheinheimera, Pseudomonas, Ensifer*, and *Pseudoxanthomonas*, were detected for the treatment by the organic soil, in contrast to *Microvirga* (Figure 3C). Two other Acidobacterial subgroups (*Gp5* and *Gp22*) as well as *Ignavibacterium, Zavarzinella*, and *Levilinea* were more abundant in the organic soil treatment, in contrast to Acidobacteria Gp4 (Figure 3C). The highest abundance of *Flavobacterium* was frequently detected in the treatment based on soil from the conventional farming system, except at T4 (Figure 3C).

### *In vitro* Antagonists of *P. capsici* in the Rhizosphere of Healthy Pepper Plants

**FIGURE 4:**
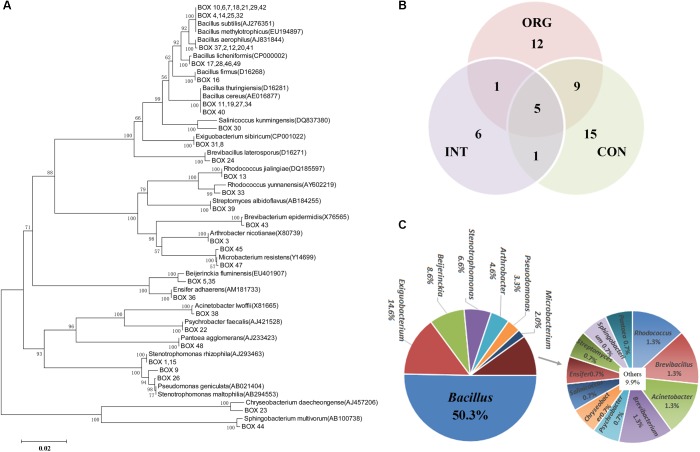
**(A)** Phylogenetic analysis of 16S rRNA gene of antagonists in each BOX-PCR pattern, number on the nodes = (bootstrap value/1000) × 100; **(B)** Venn plot showing numbers of *in vitro* antagonists with unique BOX-PCR pattern among treatments by soils from the organic (ORG), integrated (INT) and conventional (CON) farming systems. **(C)** Percentage of *in vitro* antagonists affiliated to different genera.

Rhizospheric samples from healthy plants were analyzed by a cultivation-dependent approach. A total of 1,215 bacterial isolates were acquired. Among them, 151 isolates showed *in vitro* antagonistic activities against *P. capsici*. BOX-PCR analysis further assigned these antagonists into 49 patterns (Figure 4A and Supplementary Table S3), with only five patterns shared among the different treatments (Figure 4B). Most patterns were specifically detected in each treatment (Figure 4B). The 16S *rRNA* amplicons of representative isolates for each BOX-PCR pattern were subjected to Sanger sequencing analysis. Altogether, these *in vitro* antagonists were assigned into 18 genera, with most of them affiliated to the following taxa: *Bacillus (*76 isolates, 50.3%), *Exiguobacterium* (22 isolates, 14.6%), *Beijerinckia* (13 isolates, 8.6%), *Stenotrophomonas* (6.6%), *Arthrobacter* (4.6%), and *Pseudomonas* (3.3%) (Figure 4C). Phylogenetic analysis further indicated that 16S *rRNA* genes of isolates from 26 BOX-PCR patterns were highly similar to *B. subtilis* (AJ276351), *B. methylotrophicus* (EU194897), *B. thuringiensis* (D16281), *B. cereus* (AE016877), *B. licheniformis* (CP000002), *B. aerophilus* (AJ831844), and *B. firmus* (D16268) (Figure 4A). Isolates of one BOX-PCR pattern shared high similarity with *Pseudomonas geniculate* (AB021404) (Figure 4A). Isolates of seven BOX-PCR patterns were similar to Actinobacteria strains, a phylum known to harbor antibiotic-producing bacteria (Figure 4A). In summary, these results suggested that there was a rich diversity of *in vitro* antagonists, mainly affiliated to *Bacillus, Pseudomonas*, and Actinobacteria, in the rhizosphere of healthy pepper plants.

### Enrichment of *in vitro* Antagonists Affiliated to *Bacillus* in the Rhizospheric Microbial Community

**FIGURE 5:**
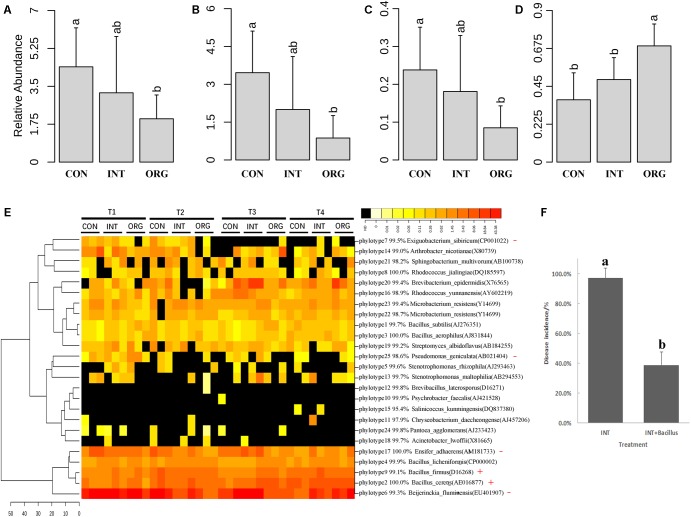
Average relative abundance of reads mapped against *in vitro* antagonists **(A)** and those antagonists affiliated to *Rhizobium* **(B)**; *Ensifer* **(C)**; and *Bacillus* **(D)**. **(E)** Heatmap analysis of *in vitro* antagonist belonging to each phylotype. Phylotype with significantly higher or lower relative abundance in the treatment by the organic soil was indicated by red cross (+) or minus (–), respectively. **(F)** Percentage (mean ± SD, *N* = 6) of pepper seedlings with *Phytophthora* blight (*P. capsici*) symptoms by soils from integrated farming system (INT) alone and inoculated with *Bacillus* consortium (INT + *Bacillus*) (*p* < 0.05, Duncan’s multiple range test). Significant differences are indicated by different letters.

To estimate the prevalence of these antagonists in the rhizosphere, 16S *rRNA* genes of antagonists were mapped against 16S r*RNA* sequence libraries. Twenty-five unique phylotypes were retrieved from subsequences (between 515F and 909R) of the 16S *rRNA* gene of representative isolates of 49 BOX-PCR patterns. All phylotypes were mapped against 16S r*RNA* sequence libraries. In all, 19,923 reads in 16S r*RNA* sequence libraries were mapped, accounting for 2.5 to 8.7% of the total reads in each library. The average relative abundance of reads that were mapped against *in vitro* antagonists was only 2.0% in the organic soil treatment, which was significantly lower than that from either the integrated or the conventional farming system (Figure 5A). Similar trends were observed for antagonists affiliated to *Rhizobium* and *Ensifer* (Figure 5B,C). Interestingly, the relative abundance of *Bacillus* was significantly higher in the soil from the organic farming system (Figure 5D). Further analysis revealed that two phylotypes (*B. cereus* and *B. firmus*) were significantly more abundant in the treatment in organic soil than in the soil from either the integrated or the conventional farming system (Figure 5E). The phylotype similar to *B. cereus* (AE016877) corresponded to four BOX-PCR patterns (11, 19, 27 and 34) (Figure 4A). The other phylotype similar to *B. firmus* (D16268) corresponded only to BOX-PCR pattern 16 (Figure 4A). As the lowest disease incidence was observed in the treatment with soil from the organic farming system, these results suggested that antagonists affiliated to *Bacillus* were possibly involved in the suppression of pepper blight in this case. A microbial consortium consisting of 18 *Bacillus* antagonists was evaluated for its ability to improve the suppressiveness of soil from the integrated farming system to pepper blight disease. During the two experiments described above, the highest disease incidence was detected in the soil from the integrated farming system. Here, the extent of disease incidence under treatment of the soil with the *Bacillus* consortium was only 38.9%, which was significantly lower than the treatment with soil from the integrated farming system alone (97.2%) (Figure 5F). These results indicated that these *Bacillus* antagonists effectively improved the ability of the soil from the integrated farming system for suppressing pepper blight disease.

## Discussion

This study was based on a long-term greenhouse experiment in which several agricultural management schemes, including crop rotation, tillage, and irrigation, were the same for all three farming systems since 2002. The largest difference among the three farming systems lies in fertilization, pest and plant disease control. This comparatively long-term experiment is believed to attenuate unexpected effects, such as initial differences among soils (Schreiter et al., 2014; Schlemper et al., 2017), plants (Weinert et al., 2011), and management strategies (Hartmann et al., 2014). As compared to the conventional farming system, disease severities were lower under the organic farming system (Yang et al., 2009a,b). Thus, this comparative experiment might provide an opportunity to study the mechanisms of soil microorganisms on plant health, which are prerequisite for engineering the soil microbiome for the suppression of soil-borne plant diseases. Here, we used complementary methods, including growth-chamber experiments, high throughput sequencing, and cultivation-dependent analysis to study the microbial diversity associated with soil suppression of pepper blight disease. Our study provides a new insight on the mechanics of disease suppression by a soil forged through long-term organic farming.

### Agricultural Management and Suppression of Soil-Borne Diseases

The soil microbiome in the organic farming system demonstrated a greater ability to suppress pepper blight disease. Similar results were observed for other soil-borne diseases in organic farms (Wang et al., 2001; Champeil et al., 2004; Postma et al., 2008; Lenc et al., 2015). In general, natural soils can suppress soil-borne plant diseases, as evidenced by the observation that plants grown in sterilized soils are more susceptible to pathogens than those growing in non-sterilized soils (Thuerig et al., 2009; Mendes et al., 2011). Such soil suppressiveness has been attributed to the activities of indigenous soil microorganisms. Under organic farming systems, several agricultural management practices, such as the utilization of organic fertilizer (Sharma et al., 2012; Wang et al., 2015), intercropping (Lithourgidis et al., 2011; Boudreau, 2013), and crop rotation (Ball et al., 2005), were employed to preserve the well-being of agroecosystems, and some of these procedures actually enhanced the ability of the soil microbiome to suppress plant pathogens (van Bruggen and Finckh, 2016). Among them, the use of compost contributed to the suppression of several plant diseases caused by *Pythium ultimum*, *Rhizoctonia solani*, *Verticillium dahlia*, *Fusarium oxysporum*, *Ralstonia solanacearum*, and *P. capsici* (Noble and Coventry, 2010; Sang et al., 2010; Liu et al., 2016). In the present study, a large amount (approximately 165 t ha^−1^) of compost has been applied annually in the organic farming system since 2002. The suppression of *Phytophthora* blight diseases by compost is well established (Chae et al., 2006; Sang et al., 2010; Hadar and Papadopoulou, 2012). The mechanism whereby compost has such a marked effect on disease suppression was assumed to be multidimensional: nutrient supplementation, antibiotics, antagonists, and alteration of soil physicochemical properties (Noble and Coventry, 2010). Here, the influence of soil physicochemical properties was mitigated by growing pepper in a mixture consisting largely of sand, except for a small fraction of soil used as an inoculant of the microbiome. Such a procedure may allow us to discern the effect of the soil microbiome on plant health. Nonetheless, it is worth noting that soil-disease suppressiveness under long-term organic farming is highly complex, and that in its inherent limitations, this simplified procedure does not allow full understanding of the complex interactions among soil physicochemical properties, plants, and the microbiome. Both physicochemical soil properties and plants likely influence the structure and function of the soil microbial community (Babin et al., 2012; Ding et al., 2013; Pronk et al., 2017). Nevertheless, the results reported herein indicate that changes in the soil microbial community associated with long-term organic farming likely contributed to soil suppressiveness to pepper blight caused by *P. capsici*.

### *Bacillus* and *Phytophthora* Blight Suppression

Recently, soil suppressiveness to soil-borne diseases was attributed to collective changes in soil or rhizospheric microbial community (Yim et al., 2015; Cha et al., 2016; Xiong et al., 2017; Gouda et al., 2018), which are extremely complex, frequently with many thousands of different OTUs simultaneously. Bacterial diversity analysis either by high throughput sequencing technologies (Yim et al., 2015) or DNA microarray (Mendes et al., 2011) allows an in-depth analysis of these microbial communities. Here, genera such as *Bacillus*, Acidobacteria (*Gp22, Gp16, GP6*) and *Ignavibacterium* were more abundant in the rhizosphere of pepper treated by the organic soil. Among them, *Bacillus* was likely associated with the suppression of pepper blight disease. Cultivation-dependent studies provided evidence that most (50%) *in vitro* antagonists from the rhizosphere of pepper free of disease symptoms were affiliated to *Bacillus*, and their abundance was higher in the organic soil treatment than in the other treatments under study. The other line of evidence is that a microbial consortium of 18 *Bacillus* antagonists isolated from pepper plants free of any disease symptoms was able to enhance the ability of the soil from the integrated farming system to suppress pepper blight. Strains of *Bacillus* such as *B. subtilis* Bs 8B-1 (Khabbaz et al., 2015), *B. licheniformis* ATCC 14580 (Bibi et al., 2018) and *B. cereus* B1301 (Yang et al., 2012) were able to suppress *Phytophthora* blight under both greenhouse and field conditions. Previously, some agricultural management practices associated with organic farming, such as amendment with organic materials, were able to enhance soil suppressiveness against several plant diseases (van Bruggen et al., 2016). This phenomenon is often referred to as general suppressiveness, whereby the activities of soil indigenous microorganisms were thought to be triggered non-specifically (van Bruggen and Finckh, 2016). Altogether, our results demonstrate that specific enrichment of *Bacillus* in the rhizosphere of pepper contributed to the suppression of pepper blight in soil subjected to long-term organic farming.

Members of Acidobacteria were assumed as K-strategists that may respond to environmental perturbation slowly (Fierer et al., 2007). *Ignavibacterium* was suggested to be involved in the degradation of organic matter (Bleyen et al., 2018), which was much higher in the soil under the organic farming. Thus, it is possible that a high abundance of Acidobacteria (*Gp22, Gp16, GP6*) and *Ignavibacterium* may be associated with the long-term use of compost, which has been extensively degraded by microorganisms. Thus, the roles of these genera on the suppression of pepper blight disease require further study. *In vitro* antagonists affiliated to Alpha- and Gamma-Proteobacteria were associated with specific suppressiveness in soils (Mendes et al., 2011; Cha et al., 2016). Here, their abundance was lower in the treatment with organic soil than in the soil from either the integrated or the conventional farming system. This result suggests that these bacteria contributed less to the suppression of pepper blight in this experiment. However, further analysis, such as meta-transcriptomic or meta-proteomic studies, may shed more light into the functions of different microbial taxa in the rhizosphere. General suppressiveness differs from specific suppressiveness in several aspects, such as the spectrum and strength of disease suppression, conductivity, or development of disease suppression (van Bruggen and Finckh, 2016). Thus, it is possible that general and specific suppressiveness are related to different microorganisms. However, this is still a case study and the knowledge acquired needs to be validated in other long-term experiments to acquire a comprehensive understanding of the role of the soil microbiome in plant health, as it is mainly shaped by each specific environmental niche (Bahram et al., 2018).

## Conclusion

The soil microbiome in the organic farming system was more effective in suppressing pepper blight disease than those either in the integrated or the conventional farming system and resultedin a different microbial assemblage in the rhizosphere of pepper seedlings. Analyses of *in vitro* antagonists, rhizospheric microbial communities and microbial consortia highlight the role of *Bacillus* on the suppression of pepper blight.

## Author Contributions

JL, G-cD, TX, and HL designed the experiments, wrote the manuscript, and analyzed the data. HL, XC, and JG performed the experiments. All authors reviewed the manuscript.

## Conflict of Interest Statement

The authors declare that the research was conducted in the absence of any commercial or financial relationships that could be construed as a potential conflict of interest.

## Footnotes

1 http://www.r-project.org/

2 www.freebioinfo.org

3 https://galaxyproject.org/

## References

[B1] Adesina M. F. Lembke A. Costa R. Speksnijder A. Smalla K. (2007). Screening of bacterial isolates from various European soils for in vitro antagonistic activity towards *Rhizoctonia solani* and *Fusarium oxysporum*: site-dependent composition and diversity revealed. *Soil Biol. Biochem.* 39 2818–2828. 10.1016/j.soilbio.2007.06.004

[B2] Babin D. Ding G. Pronk G. J. Heister K. Kögel-Knabner I. Smalla K. (2012). Metal oxides, clay minerals and charcoal determine the composition of microbial communities in matured artificial soils and their response to phenanthrene. *FEMS Microbiol. Ecol.* 86 3–14. 10.1111/1574-6941.12058 23336569

[B3] Bahram M. Hildebrand F. Forslund S. K. Anderson J. L. Soudzilovskaia N. A. Bodegom P. M. (2018). Structure and function of the global topsoil microbiome. *Nature* 560 233–243. 10.1038/s41586-018-0386-6 30069051

[B4] Ball B. C. Bingham I. Rees R. M. Watson C. A. Litterick A. (2005). The role of crop rotations in determining soil structure and crop growth conditions. *Can. J. Soil Sci.* 85 557–577. 10.4141/S04-078

[B5] Barberán A. Bates S. T. Casamayor E. O. Fierer N. (2012). Using network analysis to explore co-occurrence patterns in soil microbial communities. *ISME J.* 6 343–351. 10.1038/ismej.2011.119 21900968 PMC3260507

[B6] Barchenger D. W. Lamour K. H. Sheu Z. M. Shrestha S. Kumar S. Lin S. (2017). Intra- and intergenomic variation of ploidy and clonality characterize *Phytophthora capsici* on *Capsicum* sp. in Taiwan. *Mycol. Prog.* 16 955–963. 10.1007/s11557-017-1330-0

[B7] Bastian M. Heymann S. Jacomy M. (2009). “Gephi: An open source software for exploring and manipulating networks,” in *Proceedings of the International AAAI conference on weblogs and social media*, San Jose, CA.

[B8] Bi Y. Hu J. Cui X. Shao J. Lu X. Meng Q. (2014). Sexual reproduction increases the possibility that *Phytophthora capsici* will develop resistance to dimethomorph in China. *Plant Pathol.* 63 1365–1373. 10.1111/ppa.12220

[B9] Bibi F. Strobel G. A. Naseer M. I. Yasir M. Khalaf Al-Ghamdi A. A. Azhar E. I. (2018). Microbial flora associated with the halophyte–salsola imbricate and its biotechnical potential. *Front. Microbiol.* 9:65 10.3389/fmicb.2018.00065 PMC5797760 29445362

[B10] Bleyen N. Hendrix K. Moors H. Durce D. Vasile M. Valcke E. (2018). Biodegradability of dissolved organic matter in boom clay pore water under nitrate-reducing conditions: effect of additional C and P sources. *Appl. Geochem.* 92 45–58. 10.1016/j.apgeochem.2018.02.005

[B11] Boudreau M. A. (2013). Diseases in intercropping systems. *Ann. Rev. Phytopathol.* 51 499–519. 10.1146/annurev-phyto-082712-102246 23725470

[B12] Caporaso J. G. Kuczynski J. Stombaugh J. Bittinger K. Bushman F. D. Costello E. K. (2010). QIIME allows analysis of high-throughput community sequencing data. *Nat. Methods* 7 335–336. 10.1038/nmeth.f.303 20383131 PMC3156573

[B13] Cha J. Y. Han S. Hong H. J. Cho H. Kim D. Kwon Y. (2016). Microbial and biochemical basis of a *Fusarium* wilt-suppressive soil. *ISME J.* 10 119–129. 10.1038/ismej.2015.95 26057845 PMC4681868

[B14] Chae D. H. De Jin R. Hwangbo H. Kim Y. W. Kim Y. C. Park R. D. (2006). Control of late blight (*Phytophthora capsici*) in pepper plant with a compost containing multitude of chitinase-producing bacteria. *Biocontrol* 51 339–351. 10.1007/s10526-005-2934-x

[B15] Champeil A. Fourbet J. F. Doré T. Rossignol L. (2004). Influence of cropping system on *Fusarium* head blight and mycotoxin levels in winter wheat. *Crop Prot.* 23 531–537. 10.1016/j.cropro.2003.10.011

[B16] Cole J. R. Wang Q. Fish J. A. Chai B. McGarrell B. M. Sun Y. (2014). Ribosomal database project: data and tools for high throughput rRNA analysis. *Nucleic Acids Res.* 42 633–642. 10.1093/nar/gkt1244 24288368 PMC3965039

[B17] Ding G. Heuer H. Smalla K. (2012). Dynamics of bacterial communities in two unpolluted soils after spiking with phenanthrene: soil type specific and common responders. *Front. Microbiol.* 3:290. 10.3389/fmicb.2012.00290 22934091 PMC3423926

[B18] Ding G. Heuer H. Zuhlke S. Spiteller M. Pronk G. J. Heister K. (2010). Soil type-dependent responses to phenanthrene as revealed by determining the diversity and abundance of polycyclic aromatic hydrocarbon ring-hydroxylating dioxygenase genes by using a novel PCR detection system. *Appl. Environ. Microbiol.* 76 4765–4771. 10.1128/AEM.00047-10 20495045 PMC2901735

[B19] Ding G. Pronk G. J. Babin D. Heuer H. Heister K. Kögel-Knabner I. (2013). Mineral composition and charcoal determine the bacterial community structure in artificial soils. *FEMS Microbiol. Ecol.* 86 15–25. 10.1111/1574-6941.12070 23289489

[B20] Fan B. Wang C. Song X. Ding X. Wu L. Wu H. (2018). *Bacillus velezensis* FZB42 in 2018: the gram-positive model strain for plant growth promotion and biocontrol. *Front. Microbiol.* 9:2491. 10.3389/fmicb.2018.02491 30386322 PMC6198173

[B21] Fernandez A. L. Sheaffer C. C. Wyse D. L. Staley C. Gould T. J. Sadowsky M. J. (2016). Associations between soil bacterial community structure and nutrient cycling functions in long-term organic farm soils following cover crop and organic fertilizer amendment. *Sci. Total Environ.* 566-567 949–959. 10.1016/j.scitotenv.2016.05.073 27288977

[B22] Fierer N. Bradford M. A. Jackson R. B. (2007). Toward an ecological classification of soil bacteria. *Ecology* 88 1354–1364. 10.1890/05-1839 17601128

[B23] Gómez Expósito R. de Bruijn I. Postma J. Raaijmakers J. M. (2017). Current insights into the role of rhizosphere bacteria in disease suppressive soils. *Front. Microbiol.* 8:2529. 10.3389/fmicb.2017.02529 29326674 PMC5741648

[B24] Gouda S. Kerry R. G. Das G. Paramithiotis S. Shin H. S. Patra J. K. (2018). Revitalization of plant growth promoting rhizobacteria for sustainable development in agriculture. *Microbiol. Res.* 206 131–140. 10.1016/j.micres.2017.08.016 29146250

[B25] Hadar Y. Papadopoulou K. K. (2012). Suppressive composts: microbial ecology links between abiotic environments and healthy plants. *Ann. Rev. Phytopathol.* 50 133–153. 10.1146/annurev-phyto-081211-172914 22920558

[B26] Han H. Teng Y. Yang H. Li J. (2017). Effects of long-term use of compost on N_2_O and CO_2_ fluxes in greenhouse vegetable systems. *Compost Sci. Uti.* 25(Suppl. 1), S61–S69. 10.1080/1065657X.2016.1238786

[B27] Hartmann M. Frey B. Mayer J. Mader P. Widmer F. (2014). Distinct soil microbial diversity under long-term organic and conventional farming. *ISME J.* 9 1177–1194. 10.1038/ismej.2014.210 25350160 PMC4409162

[B28] Hothorn T. Bretz F. Westfall P. (2017). Simultaneous inference in general parametric models. *Biomed. J.* 50 346–363. 10.1002/bimj.200810425 18481363

[B29] Khabbaz S. E. Zhang L. Cáceres L. A. Sumarah M. Wang A. Abbasi P. A. (2015). Characterisation of antagonistic *Bacillus* and *Pseudomonas* strains for biocontrol potential and suppression of damping-off and root rot diseases. *Ann. Appl. Biol.* 166 456–471. 10.1111/aab.12196

[B30] Kropf S. Läuter J. Eszlinger M. Krohn K. Paschke R. (2004). Nonparametric multiple test procedures with data-driven order of hypotheses and with weighted hypotheses. *J. Stat. Plan. Inference* 125 31–47. 10.1016/j.jspi.2003.07.021

[B31] Lane D. J. (1991). “16S/23S rRNA Sequencing,” in *Nucleic Acid Techniques in Bacterial Systematics*, eds Stackebrandt E. Goodfellow M. (New York, NY: Wiley).

[B32] Lenc L. Kwaśna H. Sadowski C. Grabowski A. (2015). Microbiota in wheat roots, rhizosphere and soil in crops grown in organic and other production systems. *J. Phytopathol.* 163 245–263. 10.1111/jph.12313

[B33] Li F. Chen L. Zhang J. Yin J. Huang S. (2017). Bacterial community structure after long-term organic and inorganic fertilization reveals important associations between soil nutrients and specific taxa involved in nutrient transformations. *Front. Microbiol.* 8:187. 10.3389/fmicb.2017.00187 28232824 PMC5298992

[B34] Lithourgidis A. S. Dordas C. A. Damalas C. A. Vlachostergios D. N. (2011). Annual intercrops: an alternative pathway for sustainable agriculture. *Aust. J. Crop Sci.* 5 396–410.

[B35] Liu H. Xiong W. Zhang R. Hang X. Wang D. Li R. (2018). Continuous application of different organic additives can suppress tomato disease by inducing the healthy rhizospheric microbiota through alterations to the bulk soil microflora. *Plant Soil.* 423 229–240. 10.1007/s11104-017-3504-6

[B36] Liu L. Sun C. He X. Liu X. Wu H. Liu M. (2016). The secondary compost products enhances soil suppressive capacity against bacterial wilt of tomato caused by *Ralstonia solanacearum*. *Eur. J. Soil Biol.* 75 70–78. 10.1016/j.ejsobi.2016.04.005

[B37] Louws F. J. Fulbright D. W. Stephens C. T. De Bruijn F. J. (1994). Specific genomic fingerprints of phytopathogenic *Xanthomonas* and *Pseudomonas* pathovars and strains generated with repetitive sequences and PCR. *Appl. Environ. Microbiol.* 60 2286–2295. 8074510 10.1128/aem.60.7.2286-2295.1994 PMC201645

[B38] Luo P. Han X. Wang Y. Han M. Shi H. Liu N. (2015). Influence of long-term fertilization on soil microbial biomass, dehydrogenase activity, and bacterial and fungal community structure in a brown soil of northeast China. *Ann. Microbiol.* 65 533–542. 10.1007/s13213-014-0889-9 25705148 PMC4331610

[B39] Marschner P. Kandeler E. Marschner B. (2003). Structure and function of the soil microbial community in a long-term fertilizer experiment. *Soil Biol. Biochem.* 35 453–461. 10.1007/s00248-008-9372-0 18347845

[B40] Mendes R. Kruijt M. de Bruijn I. Dekkers E. van der Voort M. Schneider J. H. M. (2011). Deciphering the rhizosphere microbiome for disease-suppressive bacteria. *Science* 332 1097–1100. 10.1126/science.1203980 21551032

[B41] Michelsen C. F. Watrous J. Glaring M. A. Kersten R. Koyama N. Dorrestein P. C. (2015). Nonribosomal peptides, key biocontrol components for *Pseudomonas fluorescens* In5, isolated from a Greenlandic suppressive soil. *mBio* 6:e00079. 10.1128/mBio.00079-15 25784695 PMC4453515

[B42] Ndubuisi-Nnaji U. U. Adegoke A. A. Ogbu H. I. Ezenobi N. O. Okoh A. I. (2011). Effect of long-term organic fertilizer application on soil microbial dynamics. *Afr. J. Biotechnol.* 10 556–559. 10.5897/ajb10.699

[B43] Noble R. Coventry E. (2010). Suppression of soil-borne plant diseases with composts: a review. *Biocontrol Sci. Technol.* 15 3–20. 10.1080/09583150400015904 24373678

[B44] Paidhungat M. Setlow B. Driks A. Setlow P. (2000). Characterization of spores of *Bacillus subtilis* which lack dipicolinic acid. *J. Bacteriol.* 182 5505–5512. 10.1128/JB.182.19.5505-5512.2000 10986255 PMC110995

[B45] Postma J. Schilder M. T. Bloem J. van Leeuwen-Haagsma W. K. (2008). Soilsuppressiveness and functional diversity of the soil microflora in organic farming systems. *Soil Biol. Biochem.* 40 2394–2406. 10.1016/j.soilbio.2008.05.023

[B46] Pronk G. J. Heister J. Vogel C. Babin D. Bachmann J. Ding G. (2017). Interaction of minerals, organic matter, and microorganisms during biogeochemical interface formation as shown by a series of artificial soil experiments. *Biol. Fertil. Soils* 53 9–22. 10.1007/s00374-016-1161-1

[B47] Qiu M. Zhang R. Xue C. Zhang S. Li S. Zhang N. (2012). Application of bio-organic fertilizer can control *Fusarium* wilt of cucumber plants by regulating microbial community of rhizosphere soil. *Biol. Fertil. Soils* 48 807–816. 10.1007/s00374-012-0675-4

[B48] Rognes T. Flouri T. Nichols B. Quince C. Mahe F. (2016). VSEARCH: a versatile open source tool for metagenomics. *PeerJ* 4:e2584. 10.7717/peerj.2584 27781170 PMC5075697

[B49] Sang M. K. Kim J. G. Kim K. D. (2010). Biocontrol activity and induction of systemic resistance in pepper by compost water extracts against *Phytophthora capsici*. *Phytopathology* 100 774–783. 10.1094/PHYTO-100-8-0774 20626281

[B50] Schlemper T. R. Leite M. F. A. Lucheta A. R. Shimels M. Bouwmeester H. J. van Veen J. A. (2017). Rhizobacterial community structure differences among sorghum cultivars in different growth stages and soils. *FEMS Microbiol. Ecol.* 93 1–11. 10.1093/femsec/fix096 28830071

[B51] Schloss P. D. Westcott S. L. Ryabin T. Hall J. R. Hartmann M. Hollister E. B. (2009). Introducing mothur: open-source, platform-independent, community-supported software for describing and comparing microbial communities. *Appl. Environ. Microbiol.* 75 7537–7541. 10.1128/AEM.01541-09 19801464 PMC2786419

[B52] Schreiter S. Ding G. Heuer H. Neumann G. Sandmann M. Grosch R. (2014). Effect of the soil type on the microbiome in the rhizosphere of field-grown lettuce. *Front. Microbiol.* 5:144 10.3389/fmicb.2014.00144 PMC3986527 24782839

[B53] Sharma K. Bruns C. Butz A. F. Finckh M. R. (2012). Effects of fertilizers and plant strengtheners on the susceptibility of tomatoes to single and mixed isolates of *Phytophthora infestans*. *Eur. J. Plant Pathol.* 133 739–751. 10.1007/s10658-012-9954-z

[B54] Shen Z. Ruan Y. Chao X. Zhang J. Li R. Shen Q. (2015). Rhizosphere microbial community manipulated by 2 years of consecutive biofertilizer application associated with banana *Fusarium* wilt disease suppression. *Biol. Fertil. Soils* 51 553–562. 10.1007/s00374-015-1002-7

[B55] Strunnikova O. K. Vishnevskaya N. A. Ruchiy A. S. Shakhnazarova V. Y. Vorobyov N. A. Chebotar V. K. (2015). The influence of soils with different textures on development, colonization capacity and interactions between *Fusarium culmorum* and *Pseudomonas fluorescens* in soil and on barley roots. *Plant Soil.* 389 131–144. 10.1007/s11104-014-2351-y

[B56] Tamura K. Stecher G. Peterson D. Filipski A. Kumar S. (2013). MEGA6: molecular evolutionary genetics analysis version 6.0. *Mol. Biol. Evol.* 30 2725–2729. 10.1093/molbev/mst197 24132122 PMC3840312

[B57] Thuerig B. Fließbach A. Berger N. Fuchs J. G. Kraus N. Mahlberg N. (2009). Re-establishment of suppressiveness to soil- and air-borne diseases by re-inoculation of soil microbial communities. *Soil Biol. Biochem.* 41 2153–2161. 10.1016/j.soilbio.2009.07.028

[B58] van Bruggen A. H. C. Finckh M. R. (2016). Plant diseases and management approaches in organic farming systems. *Ann. Rev. Phytopathol.* 54 25–54. 10.1146/annurev-phyto-080615-100123 27215969

[B59] van Bruggen A. H. C. Gamliel A. Finckhc M. R. (2016). Plant disease management in organic farming systems. *Pest Manag. Sci.* 72 30–44. 10.1002/ps.4145 26331771

[B60] van der Voort M. Kempenaar M. van Driel M. Raaijmakers J. M. Mendes R. (2016). Impact of soil heat on reassembly of bacterial communities in the rhizosphere microbiome and plant disease suppression. *Ecol. Lett.* 19 375–382. 10.1111/ele.12567 26833547

[B61] Wang L. Lu X. Yuan H. Wang B. Shen Q. (2015). Application of bio-organic fertilizer to control tomato *Fusarium* wilting by manipulating soil microbial communities and development. *Commun. Soil Sci. Plant Anal.* 46 2311–2322. 10.1080/00103624.2015.1081694

[B62] Wang R. Xu H. Mridha M. A. U. (2001). Phytophthora resistance of organically-fertilized tomato plants. *J. Crop Prod.* 3 77–84. 10.1300/J144v03n01_07

[B63] Weinert N. Piceno Y. Ding G. Meincke R. Heuer H. Berg G. (2011). Phylo-Chip hybridization uncovered an enormous bacterial diversity in the rhizosphere of different potato cultivars: many common and few cultivar-dependent taxa. *FEMS Microbiol. Ecol.* 75 497–506. 10.1111/j.1574-6941.2010.01025.x 21204872

[B64] Weller D. M. Raaijmakers J. M. Gardener B. B. Thomashow L. S. (2002). Microbial populations responsible for specific soil suppressiveness to plant pathogens. *Ann. Rev. Phytopathol.* 40 309–348. 10.1146/annurev.phyto.40.030402.110010 12147763

[B65] Xiong W. Li R. Ren Y. Liu C. Zhao Q. Wu H. (2017). Distinct roles for soil fungal and bacterial communities associated with the suppression of vanilla *Fusarium* wilt disease. *Soil Biol. Biochem.* 107 198–207. 10.1016/j.soilbio.2017.01.010

[B66] Xiong W. Zhao Q. Zhao J. Xun W. Li R. Zhang R. (2015). Different continuous cropping spans significantly affect microbial community membership and structure in a vanilla-grown soil as revealed by deep pyrosequencing. *Microb. Ecol.* 70 209–218. 10.1007/s00248-014-0516-0 25391237

[B67] Yang H. Fan J. Ge Z. Shen Z. Lv R. Li J. (2009a). Main diseases and control effects of organic, integrated and conventional cultivation patterns of greenhouse tomato. *Chin. J. Eco Agric.* 17 933–937. 10.3724/SP.J.1011.2009.00933

[B68] Yang H. Fan J. Liang L. Meng Y. Zhang S. Li J. (2009b). Studies on the main diseases and control effects under organic, integrated and conventional cultivation patterns of cucumber in greenhouse. *Acia Agric. Boreali-sjnica* 24 240–245.

[B69] Yang M. Xu L. Xue Q. Yang J. Xu Q. Liu H. (2012). Screening potential bacterial biocontrol agents towards *Phytophthora capsici* in pepper. *Eur. J. Plant Pathol.* 134 811–820. 10.1007/s10658-012-0057-7

[B70] Yilmaz P. Yarza P. Gerken J. Pruesse E. Quast C. (2014). The SILVA and “All-species Living Tree Project (LTP)” taxonomic frameworks. *Nucleic Acids Res.* 42 643–648. 10.1093/nar/gkt1209 24293649 PMC3965112

[B71] Yim B. Winkelmann T. Ding G. Smalla K. (2015). Different bacterial communities in heat and gamma irradiation treated replant disease soils revealed by 16S rRNA gene analysis–contribution to improved aboveground apple plant growth? *Front. Microbiol.* 6:1224. 10.3389/fmicb.2015.01224 26635733 PMC4654428

[B72] Yin C. Hulbert S. H. Schroeder K. L. Mavrodi O. Mavrodi D. Dhingra A. (2013). Role of bacterial communities in the natural suppression of *Rhizoctonia solani* bare patch disease of wheat (*Triticum aestivum* L.). *Appl. Environ. Microbiol.* 79 7428–7438. 10.1128/AEM.01610-13 24056471 PMC3837727

[B73] Zhang N. Pan R. Shen Y. Yuan J. Wang L. Luo X. (2017). Development of a novel bio-organic fertilizer for plant growth promotion and suppression of rhizome rot in ginger. *Biol. Control* 114 97–105. 10.1016/j.biocontrol.2017.08.001

