# Supplementary material for: Long-Term Organic Farming Manipulated Rhizospheric Microbiome and Bacillus Antagonism Against Pepper Blight (Phytophthora capsici)
Source: Front Microbiol. 2019 Feb 27;10:342. doi: 10.3389/fmicb.2019.00342 (PMC6401385; doi:10.3389/fmicb.2019.00342)
Supplement: Supplementary file 1 [file Table_1.DOCX]

**TableS1: Details of 16S *rRNA* gene amplicons sequence.**

| Samples | gcover | observed Species | Chao1 | ACE | Number_of_Phylum_detected | Percent_of_unidentified_Phylum | Number_of_Class_detected | Percent_of_unidentified_Class | Number_of_Order_detected | Percent_of_unidentified_Order | Number_of_Family_detected | Percent_of_unidentified_Family | Number_of_Genus_detected | Percent_of_unidentified_Genus |
| --- | --- | --- | --- | --- | --- | --- | --- | --- | --- | --- | --- | --- | --- | --- |
| T1CON_a | 89.9 | 4086 | 7464 | 7827 | 24 | 4.2 | 62 | 6.8 | 98 | 11.6 | 193 | 15.3 | 431 | 22.8 |
| T1CON_b | 89.4 | 3225 | 6438 | 6772 | 24 | 3.3 | 59 | 5.2 | 96 | 7.9 | 190 | 10.6 | 396 | 18.2 |
| T1CON_c | 91 | 4312 | 8052 | 8328 | 23 | 3.5 | 59 | 5.5 | 98 | 9.3 | 197 | 12.6 | 440 | 20.3 |
| T1INT_a | 81.3 | 3433 | 7342 | 7911 | 26 | 5.1 | 62 | 8.3 | 100 | 14.4 | 195 | 19.3 | 424 | 28 |
| T1INT_b | 81.9 | 2989 | 6648 | 6812 | 24 | 4.6 | 59 | 7.4 | 92 | 13.7 | 179 | 18.5 | 390 | 28.5 |
| T1INT_c | 82.5 | 3218 | 7620 | 7848 | 26 | 4.1 | 62 | 6.6 | 95 | 11.3 | 190 | 15.7 | 408 | 22.7 |
| T1ORG_a | 83.6 | 4336 | 9190 | 9967 | 25 | 4.8 | 62 | 8.1 | 98 | 13.9 | 192 | 19.7 | 427 | 27.9 |
| T1ORG_b | 84.4 | 3972 | 8048 | 8518 | 27 | 5.2 | 63 | 9 | 95 | 15.1 | 190 | 21 | 414 | 28.5 |
| T1ORG_c | 83.2 | 3517 | 7866 | 8003 | 26 | 5 | 59 | 8.9 | 95 | 15.2 | 189 | 21.4 | 404 | 28.9 |
| T2CON_a | 88.8 | 3726 | 7287 | 7477 | 24 | 4.4 | 62 | 6.8 | 98 | 11.2 | 192 | 15.1 | 414 | 23 |
| T2CON_b | 85.7 | 4399 | 9960 | 10313 | 26 | 5.3 | 67 | 7.8 | 102 | 12.8 | 195 | 17.5 | 425 | 26.1 |
| T2CON_c | 91.2 | 4471 | 8128 | 8466 | 25 | 4.1 | 68 | 6.5 | 103 | 11.1 | 209 | 15.1 | 448 | 23 |
| T2INT_a | 77.2 | 2281 | 5140 | 5726 | 21 | 4.6 | 51 | 7.2 | 80 | 12.4 | 161 | 18.1 | 329 | 25.8 |
| T2INT_b | 91.2 | 8623 | 15960 | 16641 | 28 | 6.6 | 68 | 10.5 | 110 | 17.7 | 229 | 23.5 | 559 | 32.3 |
| T2INT_c | 71.4 | 2100 | 5249 | 5779 | 23 | 5.3 | 51 | 8.8 | 77 | 15.3 | 166 | 21.4 | 322 | 30 |
| T2ORG_a | 77 | 3327 | 8231 | 8463 | 23 | 5.5 | 57 | 9 | 88 | 15.7 | 177 | 22.5 | 379 | 31.1 |
| T2ORG_b | 86.9 | 5217 | 9785 | 10190 | 28 | 6.4 | 69 | 11.2 | 102 | 19.1 | 206 | 26.3 | 467 | 34.7 |
| T2ORG_c | 83.7 | 5116 | 11218 | 11502 | 26 | 6.3 | 67 | 10.6 | 104 | 19.2 | 205 | 26.6 | 459 | 35.9 |
| T3CON_a | 86.9 | 4086 | 7946 | 8130 | 23 | 6 | 63 | 9.2 | 102 | 15.4 | 200 | 20.8 | 430 | 29.6 |
| T3CON_b | 84.7 | 3532 | 6633 | 7133 | 24 | 6 | 62 | 9.7 | 97 | 16.2 | 186 | 20.7 | 392 | 29.4 |
| T3CON_c | 90.2 | 2570 | 4749 | 4940 | 21 | 9.1 | 53 | 14.8 | 86 | 21.4 | 168 | 28.4 | 317 | 34.1 |
| T3INT_a | 88.3 | 4566 | 9172 | 9325 | 27 | 4.6 | 63 | 7.2 | 97 | 11.7 | 200 | 16.6 | 449 | 24.2 |
| T3INT_b | 80.7 | 2708 | 6018 | 6278 | 25 | 5.4 | 57 | 8.3 | 86 | 14 | 173 | 18.8 | 362 | 28 |
| T3INT_c | 87.8 | 4003 | 8438 | 8723 | 24 | 3.8 | 59 | 5.9 | 89 | 10.3 | 186 | 14.3 | 422 | 21.3 |
| T3ORG_a | 73.9 | 1811 | 4213 | 4814 | 18 | 4.6 | 49 | 8.1 | 76 | 13.3 | 151 | 18.2 | 294 | 26.5 |
| T3ORG_b | 79.5 | 3549 | 8731 | 8853 | 24 | 6.2 | 61 | 10.3 | 95 | 16.5 | 187 | 22.5 | 382 | 30.8 |
| T3ORG_c | 83 | 3370 | 7363 | 7768 | 21 | 5.1 | 60 | 8.4 | 96 | 14 | 190 | 19.1 | 381 | 26.5 |
| T4CON_a | 81.2 | 3161 | 7257 | 7803 | 24 | 4.7 | 58 | 7.3 | 89 | 11.1 | 181 | 15.4 | 387 | 23 |
| T4CON_b | 77.2 | 2302 | 6117 | 6150 | 24 | 4.7 | 55 | 7.5 | 88 | 12.6 | 164 | 17.1 | 333 | 25 |
| T4CON_c | 86.8 | 4618 | 8970 | 9059 | 28 | 6 | 62 | 9.2 | 99 | 15.5 | 201 | 20.7 | 470 | 29.6 |
| T4INT_a | 88.9 | 4519 | 9631 | 10057 | 25 | 3.5 | 61 | 5.5 | 96 | 9.3 | 182 | 13 | 411 | 19.4 |
| T4INT_b | 90.3 | 4693 | 10097 | 10445 | 26 | 2.4 | 61 | 4 | 95 | 6.8 | 188 | 10.8 | 439 | 17.4 |
| T4INT_c | 85.6 | 4218 | 9909 | 10371 | 23 | 3.6 | 57 | 5.6 | 89 | 9 | 178 | 13 | 417 | 20.3 |
| T4ORG_a | 86.9 | 4942 | 10049 | 10448 | 25 | 5.8 | 57 | 9.6 | 92 | 15.1 | 200 | 20.6 | 443 | 29 |
| T4ORG_b | 80.2 | 4175 | 10533 | 10924 | 24 | 5.5 | 60 | 9.4 | 91 | 15.9 | 181 | 22.6 | 385 | 31.2 |
| T4ORG_c | 87.2 | 4604 | 9180 | 9487 | 26 | 5.1 | 60 | 8.9 | 96 | 14.8 | 193 | 20 | 435 | 27.6 |

**Table S2: Difference on rhizospheric community composition of pepper seedlings between treatments and sampling.**

|  | Bacteria | Proteobacteria | Bacteroidetes | Firmicutes | Acidobacteria | Actinobacteria |
| --- | --- | --- | --- | --- | --- | --- |
| Sampling | 3.5* | 5.3* | 4* | 3.1* | 0.7* | 1.9* |
| Treatment | 7.9* | 11.4* | 10.2* | 2.2* | 5.6* | 3.6* |
| ORG vs CON | 9.2* | 13.2* | 13.4* | 4.1* | 7.7* | 4.7* |
| ORG vs INT | 7.4* | 9.1* | 8.2* | 1.6 | 4.3* | 3.0* |
| INT vs CON | 7.3* | 12.1* | 8.9* | 0.8 | 4.8* | 3.0* |

note: * indicates significant difference (p<0.05) on community composition as revealed by 1000 times of permutation analysis. Treatments by soils from the organic (ORG), integrated (INT) and conventional (CON) farming systems.

**Table S3: *In vitro* antagonists isolated from pepper free of *Phytophthora* blight symptoms**

| BOX-PCR pattern | Phylotype | Blast result | Numbers | | |
| --- | --- | --- | --- | --- | --- |
|  |  |  | ORG | INT | CON |
| 1 | 5 | 99.6% *Stenotrophomonas rhizophila*(AJ293463) | 6 | 0 | 0 |
| 2 | 3 | 100.0% *Bacillus aerophilus*(AJ831844) | 1 | 0 | 0 |
| 3 | 14 | 99.0% *Arthrobacter nicotianae*(X80739) | 7 | 0 | 0 |
| 4 | 1 | 99.7% *Bacillus subtilis*(AJ276351) | 2 | 0 | 1 |
| 5 | 6 | 99.2% *Beijerinckia fluminensis*(EU401907) | 6 | 1 | 4 |
| 6 | 1 | 99.9% *Bacillus methylotrophicus*(EU194897) | 7 | 0 | 3 |
| 7 | 1 | 99.9% *Bacillus methylotrophicus*(EU194897) | 2 | 0 | 0 |
| 8 | 7 | 99.5% *Exiguobacterium sibiricum*(CP001022) | 8 | 2 | 9 |
| 9 | 25 | 98.6% *Pseudomonas geniculata*(AB021404) | 2 | 0 | 3 |
| 10 | 1 | 99.9% *Bacillus methylotrophicus*(EU194897) | 5 | 6 | 2 |
| 11 | 2 | 100.0% *Bacillus cereus*(AE016877) | 1 | 0 | 0 |
| 12 | 3 | 100.0% *Bacillus aerophilus*(AJ831844) | 1 | 0 | 1 |
| 13 | 8 | 100.0% *Rhodococcus jialingiae*(DQ185597) | 1 | 0 | 0 |
| 14 | 1 | 100.0% *Bacillus subtilis*(AJ276351) | 1 | 0 | 2 |
| 15 | 5 | 99.6% *Stenotrophomonas rhizophila*(AJ293463) | 0 | 1 | 0 |
| 16 | 9 | 99.1% *Bacillus firmus*(D16268) | 0 | 1 | 0 |
| 17 | 4 | 99.9% *Bacillus licheniformis*(CP000002) | 0 | 1 | 0 |
| 18 | 1 | 99.9% *Bacillus methylotrophicus*(EU194897) | 2 | 1 | 3 |
| 19 | 2 | 99.9% *Bacillus cereus*(AE016877) | 1 | 1 | 1 |
| 20 | 3 | 100.0% *Bacillus aerophilus*(AJ831844) | 2 | 1 | 2 |
| 21 | 1 | 99.9% *Bacillus methylotrophicus*(EU194897) | 1 | 1 | 0 |
| 22 | 10 | 99.9% *Psychrobacter faecalis*(AJ421528) | 1 | 0 | 0 |
| 23 | 11 | 97.9% *Chryseobacterium daecheongense*(AJ457206) | 1 | 0 | 0 |
| 24 | 12 | 99.8% *Brevibacillus laterosporus*(D16271) | 1 | 0 | 1 |
| 25 | 1 | 99.7% *Bacillus subtilis*(AJ276351) | 1 | 0 | 1 |
| 26 | 13 | 99.7% *Stenotrophomonas maltophilia*(AB294553) | 1 | 0 | 2 |
| 27 | 2 | 100.0% *Bacillus cereus*(AE016877) | 1 | 0 | 2 |
| 28 | 4 | 99.9% *Bacillus licheniformis*(CP000002) | 3 | 0 | 0 |
| 29 | 1 | 99.9% *Bacillus methylotrophicus*(EU194897) | 0 | 0 | 4 |
| 30 | 15 | 95.4% *Salinicoccus kunmingensis*(DQ837380) | 0 | 0 | 1 |
| 31 | 7 | 99.9% *Exiguobacterium sibiricum*(CP001022) | 0 | 0 | 3 |
| 32 | 1 | 99.9% *Bacillus subtilis*(AJ276351) | 0 | 0 | 1 |
| 33 | 16 | 98.9% *Rhodococcus yunnanensis*(AY602219) | 0 | 0 | 1 |
| 34 | 2 | 100.0% *Bacillus cereus*(AE016877) | 0 | 0 | 1 |
| 35 | 6 | 99.3% *Beijerinckia fluminensis*(EU401907) | 0 | 0 | 2 |
| 36 | 17 | 100.0% *Ensifer adhaerens*(AM181733) | 0 | 0 | 1 |
| 37 | 3 | 99.9% *Bacillus aerophilus*(AJ831844) | 0 | 0 | 1 |
| 38 | 18 | 99.7% *Acinetobacter lwoffii*(X81665) | 0 | 0 | 2 |
| 39 | 19 | 99.2% *Streptomyces albidoflavus*(AB184255) | 0 | 0 | 1 |
| 40 | 2 | 100.0% *Bacillus thuringiensis*(D16281) | 0 | 0 | 2 |
| 41 | 3 | 100.0% *Bacillus aerophilus*(AJ831844) | 0 | 0 | 3 |
| 42 | 1 | 99.8% *Bacillus methylotrophicus*(EU194897) | 0 | 0 | 1 |
| 43 | 20 | 99.4% *Brevibacterium epidermidis*(X76565) | 2 | 0 | 0 |
| 44 | 21 | 98.2% *Sphingobacterium multivorum*(AB100738) | 1 | 0 | 0 |
| 45 | 22 | 98.7% *Microbacterium resistens*(Y14699) | 0 | 2 | 0 |
| 46 | 4 | 99.7% *Bacillus licheniformis*(CP000002) | 0 | 1 | 0 |
| 47 | 23 | 99.4% *Microbacterium resistens*(Y14699) | 0 | 1 | 0 |
| 48 | 24 | 99.8% *Pantoea agglomerans*(AJ233423) | 1 | 0 | 0 |
| 49 | 4 | 99.4% *Bacillus licheniformis*(CP000002) | 0 | 0 | 1 |

Note: Pepper plant from treatment by soils from the organic (ORG), integrated (INT) and conventional (CON) farming systems.
